# Effects of dietary supplements on selected hematological and biochemical parameters of Pakistani athletes

**DOI:** 10.1186/s40795-018-0250-y

**Published:** 2018-12-13

**Authors:** Sarwat Jahan, Andleeb Fatima, Iftikhar Alam, Asad Ullah, Humaira Rehman, Tayyaba Afsar, Ali Almajwal, Suhail Razak

**Affiliations:** 1 Reproductive Physiology Laboratory, Department of Animal Sciences, Quaid-i-Azam University, Islamabad, Pakistan; 2 Department of Community Health Sciences, College of Applied Medical Sciences, King Saud University, Riyadh, Kingdom of Saudi Arabia; 3 Department of Biochemistry, Quaid-i-Azam university, Islamabad, Pakistan

**Keywords:** Nutrition, Antioxidants, LH, Blood count

## Abstract

**Background:**

CDC’s (Centers for Disease Control and Prevention) National Center for Health statistics recent reports have shown that an upsurge has occurred in the use of dietary supplements among age of 20 years since 1994 and this use shown regular increase. The purpose of our study was to investigate the effect of supplements on the reproductive health on male athletes in Pakistan.

**Methods:**

A total of 150 adult male with mean age of 25.78 ± 0.56 years were included in this study and divided into four groups: Non-athlete control (*n* = 57), Non supplemental athlete control (*n* = 40), Supplemental athlete group I (*n* = 28) and supplemental athlete group II (*n* = 25). Blood (10 ml) was taken from each subject. Complete blood count was performed and 5 ml of blood was centrifuged to separate plasma and then analyzed for antioxidant enzyme (CAT, POD, GR and GSH) activities, Lipid peroxidation (TBARS), electrolyte, metal (sodium, potassium and zinc) and Luteinizing hormone (LH) concentration.

**Results:**

Complete blood count results showed normal RBC, WBC, Platelets, Hemoglobin, Hematocrit, Mean corpuscular hemoglobin and Mean corpuscular hemoglobin concentration. Antioxidant enzymes (CAT, POD, GR, GSH) increased significantly in supplemental athletes as compared to control groups. Sodium and potassium showed significant increase (*p* < 0.001) in supplemental athlete group I, while TBARS also showed significant increase (*p* < 0.05) in supplemental group I and II as compared to non athlete control while non supplemental athletes showed significant increase (*p* < 0.05) in TBARS concentration as compared to non athlete control. LH concentration was found to be decreased significantly (*p* < 0.05) in supplemental group I and II as compared to control groups.

**Conclusion:**

It is therefore concluded from the present results that oxidative stress was considerably elevated in response to supplement consumption among athletes which may affect their health haematological parameters and reproductive hormones.

## Background

Dietary supplement use in the general population is increasing day by day around the globe. An estimation of 30–50% increase has occurred in the rate of energy drinks and dietary supplements among adolescents in 2010 [1]. Young athletes have focused on different kind of dietary supplements for quick results [2] which they consider secure and lawful [3].

The frequent use of these supplements is not good for health, studies have shown that continuous use of energy drinks and dietary supplements lead into neurological and cardiovascular problems [4–6].

Athletes are constrained from the use of diverse kinds of nutraceuticals which are habitually used for the treatment of different kind of complacencies as pain management [7], osteoarthritis [8, 9] and ulcerative colitis [10]. These nutraceuticals drugs are used by varoius companies in energy drinks and dietary supplements for swift effect, which does not require any pre- approval form Food and Drug administration (FDA) [11]. FDA does not confirm the quality control of manufactory process of ingredients found in the energy drinks and supplement like Ephedra, Caffeine, Amino Acids, L-tryptophan Phenylalanine, Branched Chain Amino Acids (BCAA), Vitamins and Minerals, Vitamin A, Vitamin B6, Niacin (nicotinic acid and nicotinamide) ginseng, multivitamins, taurine, and guarana [11–13]. Due to which Athletes are prone to severe health risks, like neurological, metabolic and cardiovascular problems [14–16].

Regardless of the availability of extensive information about supplement utilization from different parts of the world, limited data is accessible from Pakistan. However, even the limited number of studies shows relatively high use of supplement consumption among the Pakistani athletes. This elevated consumption may induce serious health consequences [17]. Numerous studies have also specified some adverse effects of dietary supplements usage, including cardiovascular, hematological, metabolic, and neurological problems [16] whereas, there is modest scientific data that confirms the advantageous effects of nutritional supplements in athletes [18]. Consequently, the sum of dietary supplement consumption should be constrained within the recommended dose [19]. Among the general populations, athletes are more predisposed to use these supplements as compared to other sportsmen. Nowadays, this idea has been raised in Pakistani youth and adolescent athletes. On the other hand, as the usage of supplements is more accepted by gym exercisers, serious measures should be taken about the effects of supplement consumption. Based on biochemical and physiological action of dietary supplements, this study was conducted for the purpose to assess the possible effects of some commonly used supplements by athletes on their hematological and biochemical parameters and then comparison was made with a control group.

## Methods

### Study design and subjects

The present study was conducted at Department of Animal Sciences, Faculty of Biological Sciences, Quaid-i-Azam University and the Bio-gym Islamabad. Before onset of study, the purpose of study was verbally explained to all participants and they gave informed written consent showing their wiliness to participate in the study. The study was approved by ethical committee (BAS#256) for research on human subject, Quaid-i-Azam University, Islamabad. Male volunteer athletes who were not involved in any professional sports were selected for this study. A total of 150 adult males were included in this study. These included 93 athletes and 57 non-athletes (age: 25 ± 8 years; Range: 20–35 years). All the volunteers were informed about all the procedures and outcomes of the study.

Prior to this study self-reported questionnaire was done form all the volunteers who participated in study. Personal, social, demographical and reproductive health questions were asked in the questionnaires.

The subjects were categorized into following four groups. Control athletes and non-athletes, and athletes who, in addition to exercise, voluntarily took supplements were categorized into 2 groups on the basis of type of supplements taken.

### Experimental design

- Control group I: Non athletes (*n* = 57)
- Control group II: Non-supplemental athlete group (*n* = 40)
- Supplemental athlete group (SAG) I*: (*n* = 28)
- Supplemental athlete group (SAG) II ¥: (*n* = 25)

*(SAG I) these athletes were taking supplement comprising human growth hormone = 4 IU daily, dihydrotestosterone, 1000 mg/week, Creatine = 40,000 mg daily; Glutamine = 40,000 mg daily; Branched chain amino acids = 200,000 mg protein/day.

**¥ (SAG II)** were taking supplements including dihydrotestosterone = 1000 mg/week; cytomel 13liothyronine = 0.1 mg/day; Clenbuterol = 0.02–0.1 mg/day; Caffeine, ephedrine, aspirin = 150,000 mg/day; L-arginine = 1500–3000 mg twice/day; L-carnitine = 500–1500 mg twice/day; Vanadyl sulfate = 50 mg twice/day; medium chain triglycerides = 50 mg twice/day.

### Age and body mass index (BMI)

Age was self-reported. Weight and height were measured using the standard methods (Alam et al., 2013). BMI was calculated using weight and height and reference ranges were taken from World Health Organization defining underweight as BMI < 18.50; normal weight = 18.50–24.99; overweight≥25.00; and obese ≥30.00.

### Blood collection and serum separation

A blood sample (10 ml) was anesthetically obtained from all the participants by a trained and certified technician. All the samples were centrifuged (kokusan, Japan), for 15 min at 3000 rpm to separate plasma which was transferred to eppendorf tubes and stored at − 70 °C respectively until analyzed.

### Complete blood profile

Five milliliters of blood from syringe was transferred into lavender vacutainer, containing Ethylenediaminetetraacetic acid (EDTA) as anticoagulant. Blood count was performed on an automated hematology analyzer (Medonic M16S, Sweden) at Life Care Diagnostic laboratory, Rawalpindi. Each count presented the data of white blood cell count (WBC), red blood count (RBC) platelets count (Plt), mean platelet volume (MPV), mean corpuscular volume (MCV), measurement of hemoglobin (Hgb), and measurement of hematocrit (Hct), red blood cell distribution width percentage (RDW %), mean corpuscular hemoglobin (MCH) and mean corpuscular hemoglobin concentration (MCHC).

### Plasma analysis

Plasma samples were analyzed for the determination of antioxidant enzymes activity of Catalase CAT) [20] Peroxidase (POD) [21] Glutathione Reductase (GR) [22], Reduced Glutathione (GSH) [23], lipid peroxidation (TBARS) [24] on a UV spectrophotometer (Agilent 8453) while total protein estimation was done by AMP Diagnostics reagent kit on chemistry analyzer and hormonal concentration was detected by Enzyme Immuno Assay test kit (Amgenix Inc., USA).

### Determination of electrolytes/metal in blood

Electrolytes (Na^+^ and K^+^) and Zinc in blood was determined by following the method of [25] and [26] with some modification. It involves the following steps.

### Acid digestion of serum samples

Acid digestion was done according to the following steps.

Prior to the start of acid digestion all the apparatus was soaked with 10% Nitric acid for 24 h and then washed with 69% nitric acid. Before the process of digestion pre-digestion was done in which each sample of 0.5 ml of blood was taken and 5 ml of 69% HNO_3_ was added in it until it became clear.

### Digestion

Samples were digested manually by boiling the whole blood in HNO_3_ on a hot plate at 400 °C until half of the liquid evaporated.

### Filtration

After digestion these samples were filtered through filter paper and the final volume of filtrate was made to 15 ml by adding distilled water. Samples at this stage were ready for atomic absorption spectrometry.

### Atomic absorption spectrometry

Prior to the start of atomic absorption spectrometry the following standard solutions were prepared.

Standard solution of each salt of 1000 ppm concentration was prepared by following formula.

$$ \frac{\mathrm{Molecular}\ \mathrm{weight}\ \mathrm{of}\ \mathrm{salt}\ \mathrm{x}\ 0.1=\mathrm{x}}{\mathrm{Molecular}\ \mathrm{weight}\ \mathrm{of}\ \mathrm{sample}} $$

x grams of salt were dissolved in 100 ml of distilled water to make 1000 ppm solution.

In order to calibrate the instrument, diluted solutions having concentration from 1 ppm to 50 ppm were used. The different calculations were made using the formula.

$$ {\mathrm{C}}_1{\mathrm{V}}_{1=}{\mathrm{C}}_2{\mathrm{V}}_2 $$

Where C_1_ represent concentration and V_1_ volume of stock solution of standard andC_2_ and V_2_ are the required concentration and volume respectively.

### Determination of metal concentration

For detection of different metals by Fast Atomic Absorption Spectrometry (SAAS), different lamps specific for that particular metal were used. A series of standard elements under analysis were run. Each digested sample was subjected to Acetylene flame of atomic absorbance spectrometer (Varian AA240FS, USA) and concentration of microelements was noted. These concentrations were obtained in ppm and converted into mg/l by using the following formula

$$ \mathrm{Metal}\ \mathrm{concentration}\ \left(\mathrm{mg}/\mathrm{l}\right)=\frac{\mathrm{final}\ \mathrm{volume}\ \mathrm{of}\ \mathrm{dilution}\ \mathrm{x}\ \mathrm{metal}\ \mathrm{concentration}\ \left(\mathrm{ppm}\right)}{\mathrm{Volume}\ \mathrm{of}\ \mathrm{sample}} $$

### Determination of plasma Luteinizing hormone

The kit contained antibody-coated microtitter plate coated with 96 wells, LH standard set, contains six vials (ready to use) 0, 5, 20 50, 100 and 200 mlU/ml, Enzyme conjugate reagent 12 ml, Trimethylbenzidine (TMB) substrate one bottle (ready to use) 12 ml, stop solution one bottle (ready to use) 12 ml, 50X wash buffer concentrate one bottle 15 ml.

### Principle

This test is based on a solid phase ELISA. The assay system utilizes one anti-LH antibody for solid phase (microtiter wells) immobilization and another mouse monoclonal anti-LH antibody in the antibody-enzyme (horsereddish peroxidase) conjugate solution. The test sample is allowed to react simultaneously with the antibodies resulting in the LH molecules being sandwiched between the solid phase and enzyme-linked antibodies. Unbound LH and LH enzymes conjugate is washed off by washing buffer. Upon addition of substrate, the intensity of the color is inversely proportional to the concentration of LH in the sample; a standard curve is prepared relating color intensity to the concentration of the LH.

### Procedure

The reagents provided with kit were gently mixed prior to the start of the experiment.

Desired number of wells were secured and 50ul of standard, control and specimen were dispensed into wells. 100ul of enzyme cognate was added and the samples were mixed and incubated at 37 °C for 60 mins. Washing was done of the plate 5 times with Elisa plate washer. TMB substrate 100ul was added in wells and again incubated for 20 min at 37 °C. later on stop solution 100ul was added in order to stop the solution and the absorbance was read at 450 nm on Elisa Plate reader.

### Statistical analysis

All data is presented as the mean ± standard error of mean. Data of in vivo assays recorded was analyzed by one way ANOVA carried by computer software SPSS version 16.0. *p* < 0.05 was considered significantly significant.

## Results

### Age and body mass index (BMI)

The mean age (years) and BMI (kg/m^2^) of NA control was 25.60 ± 0.59 and 23.15 ± 0.44, NSA control 25.17 ± 0.86 and 26.43 ± 0.56, SA-I 26.43 ± 0.88 and 26.84 ± 0.72 and SA-II was 25.96 ± 1.07 and 26.77 ± 0.87 as expressed in Table 1.

**Table 1:** Mean ± SEM age (years) and body mass index (kg/m^2^) of control subjects and supplemental athletes

Group	Age (years)	BMI (kg/m^2^)
NA control (*n* = 57)	25.60 ± 0.59	23.15 ± 0.44
NSA control (*n* = 40)	25.17 ± 0.86	26.43 ± 0.56
SA-I (*n* = 28)	26.43 ± 0.88	26.84 ± 0.72
SA-II (*n* = 25)	25.96 ± 1.07	25.77 ± 0.87

### Complete blood profile

No significant difference was found in WBC count, RBC count, Hb concentrations, Hematocrit percentage (Hct %), MCH MPV levels in both groups of supplemental athletes in comparison to control subjects. Platelets count showed significant increase (*P* < 0.05) in NSA control and significant decrease (*p* < 0.05) in SA-I and SA-II as compared to NA control but all groups have platelet count well within the normal range. Mean MCV showed significant increase (p < 0.05) in NSA control and SA-I as compared to NA control while no significant difference in SA-II, as compared to NA control. However MCV of all the groups is well within the normal range. Mean corpuscular hemoglobin concentration decreased significantly (*P* < 0.001) in NSA control and SA-I, SA-II as compared to subjects of NA control group. Red blood cell Distribution Width percentage (RDW %) levels were significantly high (*p* < 0.05) in NSA control as compared to NA control while no significant difference was observed in SA-I and SA-II, in comparison with NA control group (Table 2).

**Table 2:** Mean ± SEM White blood cell count (WBC), Red blood cell count (RBC), hemoglobin count (HGH), hematocrit (HCT), Mean corpuscular volume (MCV), Mean corpuscular hemoglobin (MCH), Mean corpuscular hemoglobin concentration (MCHC) and platelet count (PLT) in control subjects and supplemental athletes

	NA control (*n* = 57)	NSA control (*n* = 40)	SA-I (*n* = 28)	SA-II (*n* = 25)
RBC (10^12^/l)	5.10 ± 0.11	4.86 ± 0.11	4.99 ± 0.12	5.23 ± 0.22
WBC (10^9^/l)	7.99 ± 0.69	8.16 ± 0.5	9.84 ± 0.8	8.60 ± 0.69
PLT (10^9^/l)	235.78 ± 7.62^ab**^	256 ± 12.63^a**^	188 ± 17.31^c**^	200.88 ± 11.49^bc**^
MPV (fl)	10.08 ± 0.21	10.10 ± 0.28	10.78 ± 0.48	10.53 ± 0.38
MCV (fl)	77.82 ± 2.44^ab*^	88.47 ± ^a*^	86.49 ± 1.25^a*^	83.21 ± 1.96^ab*^
RDW (%)	14.34 ± 0.38^b*^	16.63 ± 0.79^a*^	14.46 ± 0.21^b*^	14.60 ± 0.31^b*^
Hgb (g/dl)	15.98 ± 0.43	15.83 ± 0.25	16.38 ± 0.5	16.05 ± 0.53
Hct (%)	39.54 ± 0.95	42.74 ± 1.14	43.35 ± 1.57	43.33 ± 1.37
MCH (pg)	31.50 ± 1.09	32.75 ± 0.66	32.80 ± 0.48	30.85 ± 0.91
MCHC(g/dl)	40.46 ± 0.25^a**^	37.31 ± 0.84^b**^	37.86 ± 0.51^b**^	37.03 ± 0.33^b**^

Values are expressed as Mean ± SEM. Different alphabets show that the values are significantly different while the same alphabets demonstrate that the values produce same result

* = *p* < 0.05, ** = *p* < 0.001, *** = *p* < 0.0001

^a^Value vs NA control, ^b^Value vs NSA control, ^c^Value vs SA-1

### Antioxidant enzymes

Catalase (CAT) activity was measured in all the four groups. CAT activity increased in non-supplement athletes and Supplemental athletes-I group as (P < 0.001) compared to control. While there was no significant difference found in non-athletes and supplement athletes-II group (Table 3).

**Table 3:** Mean ± SEM Plasma Catalase (CAT), Peroxidase (POD), Glutathione Reductase (GR) and Reduced Glutathione (GSH) activity in control subjects, supplemental athletes

Group	CAT (U/min)	GR (U/l)	POD (U/min)	GSH (mM/l)
NA control (*n* = 57)	1.10 ± 0.178^b**^	24.13 ± 2.15^b***^	13.43 ± 1.28^ab*^	0.05 ± 0.009
NSA control (*n* = 40)	1.77 ± 0.386^ab**^	15.83 ± 1.36^c***^	10.24 ± 1.3^b*^	0.09 ± 0.018
SA-I (*n* = 28)	2.32 ± 0.278^a**^	26.82 ± 1.9^b***^	16.14 ± 0.98^a*^	0.11 ± 0.016
SA-II (*n* = 25)	1.04 ± 0.22^b**^	42.39 ± 6.0^a***^	15.05 ± 1.09^a*^	0.38 ± 0.324

Values are expressed as Mean ± SEM. Different alphabets show that the values are significantly different while the same alphabets demonstrate that the values produce same result

* = *p* < 0.05, ** = *p* < 0.001, *** = *p* < 0.0001

^a^Value vs NA control, ^b^Value vs NSA control, ^c^Value vs SA-1

The levels of glutathione reductase were observed in all the groups and an increase in supplemental athletes-II of (*P* < 0.0001) and decrease non-supplement athletes of (P < 0.0001) were observed when compared to the non-athletes group (Table 3).

In POD levels significant decrease of (*P* < 0.05) in supplemental athletes-I and in Supplemental athletes-II was observed when compared to the control groups.

In TBARS levels significant increase of (*P* < 0.05) was observed in non-supplemental athletes groups as compared to the non-athlete control group. While in the other groups no significant difference was observed as compared to control group.

There was no significant difference observed in GSH and protein levels when compared to the control groups (Tables 3 and 4).

**Table 4:** Mean ± SEM Total protein content and thiobarbituric acid reactive substance activity in plasma of control subjects, non supplemental and supplemental athletes

Group	Total protein (g/dl)	TBARS (nM/mg protein)
NA control (*n* = 57)	6.74 ± 0.22	1.79E-08 ± 5.22E-09^c*^
NSA control (*n* = 40)	7.41 ± 0.54	6.35E-08 ± 3.24E-09^a*^
SA-I (*n* = 28)	7.87 ± 0.22	3.84E-08 ± 3.36E-09^b*^
SA-II (*n* = 25)	7.47 ± 0.19	4.28E-08 ± 5.26E-09^b*^

Values are expressed as Mean ± SEM. Different alphabets (a, b, c) show that the values are different while similar ones demonstrate values producing similar result

* = *p* < 0.05, ** = *p* < 0.001, *** = *p* < 0.000

^a^Value vs NA control, ^b^Value vs NSA control, ^c^Value vs SA-1

### Electrolytes and metals

Plasma sodium concentration was found significantly increased (*p* < 0.001) in SA-I as compared to control groups (Table 5). While significant increase in plasma Potassium concentration was observed in SA-I as compared to control groups (Table 5). However plasma Zinc levels showed no significant difference in any group in comparison with control. (Table 5).

**Table 5:** Plasma Sodium, Potassium, Zinc and luteinizing hormone (LH) levels in control subjects and supplemental athletes. Different alphabets show that the values are significantly different while same alphabets show similar results

Group	Sodium (mg/l)	Potassium (mg/l)	Zinc (mg/l)	LH (mIU/ml
NA control (*n* = 57)	3102.60 ± 113.3^b**^	298.98 ± 26.4^b**^	0.69 ± 0.03	7.2 ± 0.77^b*^
NSA control (*n* = 40)	3136.08 ± 96.6^b**^	297.23 ± 21.7^b**^	0.88 ± 0.17	7.56 ± 1.25^b*^
SA-I (*n* = 28)	4010.49 ± 159.6^a**^	798.67 ± 46^a**^	0.69 ± 0.07	3.48 ± 0.86^b*^
SA-II (*n* = 25)	3236.93 ± 90.94^b**^	390.99 ± 15^b**^	0.56 ± 0.03	4.68 ± 1.07^b*^

Values are expressed as Mean ± SEM. Different alphabets show that the values are significantly different while the same alphabets demonstrate that the values produce same result

* = *p* < 0.05, ** = *p* < 0.001, *** = *p* < 0.0001

^a^Value vs NA control, ^b^Value vs NSA control

### Plasma Luteinizing hormone (LH) concentration

Plasma Luteinizing hormone concentration (mIU/ml) displayed significant decrease (*p* < 0.05) in SA-I (3.48 ± 0.86) and SA-II (4.68 ± 1.07) as compared to NA (7.21 ± 0.77) and NSA (7.56 ± 1.25) control groups.

## Discussion

Effect of Supplements on the exercise induce oxidative stress, inflammatory response and muscle damage have been shown by multiple mechanisms in the past. Present study was designed to show the effects of commonly used dietary supplements on reproductive health of Pakistani male athletes. The results showed that the body mass index (kg/m^2^) of non-supplemental control, non-supplemental athletes control, supplemental athletes I and II was (23.15 ± 0.44, 26.43 ± 0.56, 26.84 ± 0.72 and 25.77 ± 0.87) which is higher than the body mass index of athletes in WHO global database [27].

All the parameters of blood count were normal which are in accordance with the previous study which also reported normal blood count results in subjects using sport supplements [28].

Numerous studies have shown many health benefits of exercise, where some studies have also shown that exercise upsurges the oxygen uptake which induces oxidative stress, high level of reactive oxygen species (ROS) and free radicals in the body which at times lead into the breakdown of fatty acids, amino acids, proteins and DNA macromolecules [29–32]. Recent studies have shown that the initiators of two important redox-sensitive signaling pathways including nuclear factor κ B (NF-κB) and mitogen activated protein kinase (MAPK) are due to ROS production during exercise [33].

Previous studies have reported increased antioxidant enzymes and antioxidant nutrients in response to extreme exercise [33–36]. Present study supports these findings as depicted by increased Catalase, Peroxidase, Glutathione reductase and TBARS levels in the plasma of athlete groups as compared to non-athlete controls. Reduced-glutathione (GSH) concentration also increased in NSA control, SA-I and SA-II as compared to NA control but the difference is not statistically significant.

In the present study, plasma Catalase activity of supplemental athlete group (SA-I) increased significantly (*p* < 0.001) than both control groups. These findings also strengthen previous findings that Creatine supplementation did not inhibit rise in oxidative stress markers (Catalase, Glutathione peroxidase and Superoxide dismutase) which rise due to exercise. Lawler et al., (2002) for the first time reported antioxidant activity of creatine. However, few reports suggested that Creatine supplementation in diseased populations, has been effective in reducing oxidative stress [34, 37, 38] There was no significant increase in second supplemental athlete group (SA-II) in plasma Catalase activity as compared to both the control goups, and it remained similar to non-athletes control (NA control). This Catalase suppression may be due to Vanadyl sulfate supplementation, as a previous study reported that Vanadyl sulfate administration to diabetic rats significantly decreased serum antioxidant enzyme levels, which were significantly raised by diabetes in muscle tissues.

Davies et al., (1987) measured lipid peroxidation in male Long Evans rats that exercise, increased lipid peroxidation as measured by TBARS assay. While many studies have supported Davies’ findings [39–41], as many have refuted them [42, 43]. The present study is also in agreement with Davies’ findings that exercise induces oxidative stress as indicated by the significantly increased levels of TBARS in non-supplemental athlete group (NSA control) and in both the supplemental athlete groups as compared to non-athlete control. (NA control); however in the supplemental groups (SA-I and SA-II) which along with other supplements also consumed Creatine (SA-I) and vanadyl sulfate (SA-II), the increase in TBARS concentration is slightly suppressed.

In present study, an increase in the plasma electrolyte was seen in athletes who used supplements as compared to those who do not use supplements like control group which is in relation to the previous studies [44–47] where Creatine supplementation causes electrolyte disturbances and renal damages.

Studies revealed that trace elements are also important in the enhancement of performance. Deficiency of Zinc in the body of athletes can lead to weight loss, fatigue and osteoporosis [48]. In our study, we observed normal Zinc levels in athletes who were using dietary supplements.

This study also investigated the effect of sports supplements on Luteinizing hormone level which was found to be significantly decreased in both the supplemental groups as compared to control groups. This indicates an inhibitory effect on the pituitary level. This inhibition in LH production may consequently lead to low Testosterone production and thus reproduction may be affected. However, this might be the effect of negative feedback of steroids (dihydrotestosterone) taken by the athletes. These results are in accordance with previous study in which men uses different type of supplements exhibit persistently reduced endogenous testosterone concentrations [49].

## Conclusion

Dietary supplements intake for a long period have been observed to have direct effect on health of athletes. The indiscriminate use of dietary supplements on a “just-in-case basis” is to be discouraged, and is likely to do more harm than good. For the safety of athletes dietary supplements where nutrient needs can be met by normal food shall be avoided and athletic coaches and trainers shall be educated in using natural food to meet the desired results. Supplements such as steroids and other ergogenic substances shall not be used by the athletes expect where medically indicated and where use is monitored by experts in the field.

## Abbreviation

BCAA: Branched Chain Amino Acids
CAT: Catalase
CDC: Centers for Disease Control
EDTA: Ethylenediaminetetraacetic acid
FAD: Flavin adenine dinucleotide
GR: Glutathione Reductase
GSH: Reduced Glutathione
LH: Luteinizing hormone
MAPK: Mitogen-activated protein kinase
NFkb: Nuclear factor kappa-light-chain-enhancer of activated B cells
POD: Peroxidase
ROS: Reactive oxygen species
SOD: Superoxide dismutase
TMB: Trimethylbenzidine
WBC: White blood cell
